# Factors associated with fear of falling among Saudi community-dwelling older adults: A cross-sectional study

**DOI:** 10.1097/MD.0000000000042864

**Published:** 2025-06-13

**Authors:** Rehab Abdulrhman Al Harbi, Mohammed T.A. Omar, Samiha Mohamed Ibrahim Abd Elkader, Saad A. Alhammad, Rehab F.M. Gwada

**Affiliations:** a Department of Physical Medicine and Rehabilitations, King Abdul-Aziz University Hospital, Jeddah, Saudi Arabia; b Physical Therapy Department for Surgery, Faculty of Physical Therapy, Cairo University, Giza, Egypt; c Department of Rehabilitation Health Sciences, College of Applied Medical Sciences, King Saud University, Riyadh, Saudi Arabia; d Physical Therapy Department, National Heart Institute, AL KIT KAT, Giza, Egypt.

**Keywords:** community-dwelling, falls, fear of falling, older adults

## Abstract

Fear of falling (FOF) is closely associated with increased disability among older adults, resulting in significant public health concerns. This study aimed to determine the risk factors and predictors of falling (FOF) in older adults in Saudi Arabia. This cross-sectional study recruited 170 older adults (aged ≥ 60 years) from 3 tertiary hospitals and the Geriatric Society Center in Jeddah. FOF was assessed using the Falls Efficacy Scale-International (FES-I). Data were collected using the short Geriatric Depression Scale-15 (GDS-15) and physical functional assessments, including activities of daily living questionnaire, time up and go test, and hand grip strength. The prevalence of FOF among the older Saudi adults was 46.5%. Predictors of FOF included poor health perception (OR = 10.5, 95% CI = 1.26–87.73; *P* = .03), female gender (OR = 6.17, 95% CI = 1.57–24.14; *P* = .009), vision problems (OR = 3.81, 95% CI = 1.58–9.21; *P* = .003), a history of falls (OR = 3.29, 95% CI = 1.35–8.01; *P* = .009), and the timed up and go score (OR = 1.38, 95% CI = 1.09–1.17; *P* = .007), while no medication use is more likely to have less FOF (OR = 0.03, 95% CI = 0–0.40, *P* = .007). FOF is a prevalent issue and is associated with several factors, highlighting the importance of FOF assessments among older adults in Saudi Arabia.

## 1. Introduction

Fear of falling (FOF) is closely associated with increased disability among older adults, resulting in significant public health concerns. FOF is not only a reasonable apprehension stemming from the psychological aftermath of a fall, but can also impact individuals who have not encountered a fall themselves.^[1,2]^ The prevalence of FOF varies substantially among older adults residing in the community, ranging from 21% to 85%.^[3]^ Furthermore, older adults who had experienced falls exhibited FOF prevalence ranging from 29% to 92%, while those who had not experienced falls displayed prevalence rates between 12% and 65%.^[4]^

Fear of falling is not only an immediate consequence of falls but also a substantial risk factor for future falls.^[5]^ Moreover, FOF leads to a range of adverse effects, including reduced strength, avoidance of physical activity, deconditioning, depression, physical and mental frailty,^[6]^ heightened medication usage,^[4]^ delayed rehabilitation, increased morbidity rates, reduced quality of life,^[7]^ and decreased social engagement.^[8]^ However, recent theoretical developments suggest that FOF may be protective or maladaptive against fall risk.^[9,10]^

Several risk factors for FOF have been identified, including advanced age, female sex, history of falls, balance impairments, medication usage, limitations in physical activities, self-reported health problems, cognitive impairment, depression symptoms, and living alone.^[11–13]^ Other studies have examined the relationship between physical activity and FOF in older populations, but the results were inconsistent.^[14–16]^ Furthermore, recent systematic reviews have reported conflicting findings for some risk factors, including depression, polypharmacy, and pain.^[17]^

Unfortunately, findings investigating FOF and its associated factors cannot be generalized because of diversity in lifestyle, self-perception, social constraints, and culture. Furthermore, there is limited research on FOF and its associated risk factors among older adults in the Arab world.^[18,19]^ In Saudi Arabia, the elderly population has been steadily increasing over the past few decades, owing to rising life expectancy.^[20]^ Consequently, it is imperative to understand the predictors of FOF in the older Saudi population. Therefore, this study aimed to investigate the factors associated with and predictive of FOF in community-dwelling older adults in Saudi Arabia.

## 2. Materials and methods

### 2.1. Study design and participants

A cross-sectional predictive correlation study was conducted on Saudi older adults in 3 tertiary hospitals: King Abdulaziz Hospital, King Fahad Hospital, King Abdullah Medical Center, and the Geriatric Society Center in Jeddah. The data were collected between November 2018 and March 2019.

The inclusion criteria were age 60 years or older, ability to communicate effectively, ability to walk independently or with assistive devices, willingness to participate, and having signed the informed consent form. Based on medical records and a physician’s diagnosis, patients with dementia, severe visual impairment, or vestibular impairment, and hearing and dual sensory loss were excluded. In addition, those who had undergone amputation, joint replacement, and cardiac or abdominal surgery within the last 6 months.^[21,22]^

Participants were recruited through diverse methods: posters advertisement, physician referrals, and medical record reviews. Eligible participants were then assessed based on both inclusion and exclusion criteria to determine their suitability for the study.

This study was conducted in accordance with the principles outlined in the Declaration of Helsinki. The study was approved by the Ministry of Health Ethics Committee Directorate of Health Affairs in Jeddah, under reference number No. 00969-A00635, and the ethics committee of Applied Medical Sciences College (CAMS144a-3839), King Saud University. Signed informed consent was obtained from all the participants.

The sample size was determined using G*Power software version 3.1.9.4, considering a significance level (alpha) of 0.05, power of 80%, 15 independent predictors, and effect size of 0.15 for the multiple logistic regression analysis. The sample size was estimated at 139 participants. Accounting for potential dropouts, the required sample size was increased to 170 participants.

### 2.2. Data collection and measurement

The data were collected through either self-reported or face-to-face interviews using a data screening sheet comprising 4 sections: sociodemographic data and fall history (including the history of falls, frequency, and any related injuries), health-related factors, FOF and fall efficacy, and physical functional status.

Comorbidities, including hypertension, cardiovascular disease, diabetes mellitus, visual problems, osteoarthritis, and osteoporosis, were documented for each participant. Furthermore, the total number of comorbidities and daily medication intake (excluding vitamins and dietary supplements) were recorded.

Depression was evaluated using the 15-item Arabic Geriatric Depression Scale (GDS-15), where a score of ≥5 indicated the presence of depression.^[23]^ The Arabic GDS-15 has demonstrated good validity and reliability (Cronbach alpha = 0.88).^[23]^ Participants’ perceived health status was assessed using the initial question from the Arabic version of the 12-item short form health survey (SF-12).^[24]^

The assessment of joint pain involved using a body chart depicting the front and back of a human figure. The participants were instructed to shade the areas corresponding to the joints that had been painful for most of the preceding week. Pain in the hip, knees, and ankles was grouped together as a single variable labeled “lower extremity pain” (LEP), while pain in the shoulder, elbow, and wrist was combined into another variable termed “Upper Extremity Pain” (UEP). Additionally, a separate variable was defined as low back pain.^[6]^

Fear of falling was recorded using a single-item question: “Are you afraid of falling?” This question has been addressed in several studies,^[11,25,26]^ demonstrating good test-retest reliability.^[25]^ Respondents provided their answers on a 4-point Likert scale, with options ranging from “not afraid” to “very afraid.” Two new dichotomized variables were created for the analysis: 0 and 1. Specifically, “somewhat afraid” and “not afraid” were grouped together as 0, while “fairly afraid” and “very afraid” very afraid’ were grouped as 1.^[26]^

The validated Arabic version of the Falls Efficacy Scale-International (FES-I) was also used to assess the level of concern about falls.^[27]^ The FES-I has been used to measure the FOF in several studies.^[12,28,29]^ The FES-I scale comprises 16 questions designed to gauge FOF, considering respondents’ levels of apprehension regarding falling during various social and physical activities conducted both indoors and outdoors. Each activity item was rated on a 4-point scale, with options ranging from “not at all concerned” (scored as 1) to “very concerned” (scored as 4). The total potential score on this scale ranges from 16 to 64, with higher scores indicating a greater degree of concern about falling.^[28]^

Physical function and dependency were assessed using the self-administered Arabic version of the activities of daily living (ADL) questionnaire, which is known for its high reliability (α > 0.90) and suitability for older adults.^[30]^ The total score of the questionnaire ranged from 0 to 6. A score ≤ 2 suggests severe dependency, a score between 2.5 and 5.5 indicates partial dependency, and a score of 6 signifies complete independence.^[31]^

Functional performance was assessed using the timed up and go (TUG) test. The TUG test is a reliable and valid measure for evaluating functional mobility in older adults.^[32]^ This test involves timing the process of standing up from a straight-back chair with armrests, walking 3 m at a typical pace, executing a turn, returning, and safely sitting down.

Grip strength was assessed using a Jamar hydraulic hand dynamometer (Sammons Preston, Sangamon, Los Angeles) in the dominant hand. It demonstrated both validity and reliability in measuring grip strength in older adults.^[33]^ The measurement procedure was conducted according to the guidelines of the American Society of Hand Therapists^[34]^: participants assumed a seated position with their forearm resting on a chair, their shoulder adducted, elbow flexed at 90°, forearm in a neutral position, and wrist maintained between 0° and 30° extension and between 0° and 15° ulnar deviation. Practice sessions were permitted, and participants were instructed to exert maximum effort for 5 seconds before the release. The analysis was conducted using the average of the 3 measurements.^[34]^ Instrument calibration was performed according to the manufacturer’s instructions.

### 2.3. Statistical analysis

All analyses were performed using Statistical Package for Social Sciences version 26 (IBM, Statistical Package for Social Sciences Inc., Cary). The significance level was set at *P* = .05. Demographic and health-related factors were presented as means and standard deviations or as numbers and percentages (%).

A receiver operating characteristic (ROC) curve was employed to determine the cutoff point for the FES-I based on responses to the question, “Are you afraid of falling?” Participants were subsequently categorized into 2 groups: those with and without FOF. The cutoff point was defined as the point at which the sensitivity and specificity values were maximized. To evaluate the agreement between the 2 assessment tools, the FES-I and a single question, the kappa coefficient was used. FES-I scores were obtained for further analysis.

The relationship between continuous variables (TUG test and grip strength) and FOF was assessed using a t-test. Meanwhile, the chi-square test was employed to identify any associations between FOF and categorical variables, including age, sex, education, marital status, living status, comorbidity, polypharmacy, number of falls, activity avoidance, use of walking aids, presence of depression, ADL limitations, health perception, and pain.

A binary logistic backward stepwise regression model was constructed to investigate the independent factors influencing FOF, incorporating all variables associated with FOF (*P* < .2).^[35]^ In this statistical model, the variance inflation factor is deemed acceptable when it falls below 5.^[36]^ The results are presented as odds ratios and their corresponding 95% confidence intervals (CI). The significance level was set at *P* = .05.

## 3. Results

### 3.1. Participant characteristics

**Figure 1.**
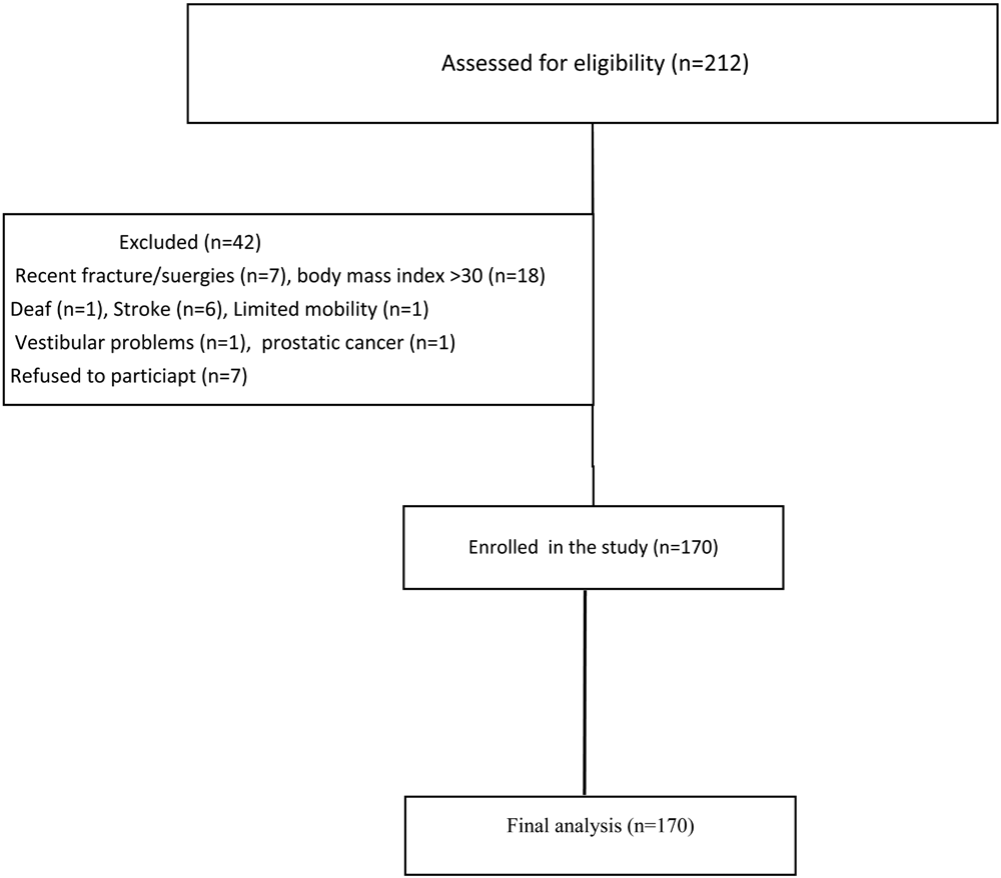
Flow chart of patient enrollments.

Initially, 212 participants underwent an eligibility assessment. Of these, 42 were excluded, leaving a total of 170 participants in the final analysis (Fig. 1). The participants had a mean age of 66.5 ± 6.9 years, with a significant majority (n = 117; 68.80%) falling within the 60 to 69 years age group. More than half of the participants (n = 93; 54.7%) were female, a substantial portion (n = 121; 71.2%) were married, and 68.2% (n = 116) had an educational background (Table 1).

**Table 1:** Demographic characteristics, and history of falls of the study participants (n = 170).

	Total (n = 170)		
		N	%
Age (yr)		66.5 ± 6.9^˄^	
	60–69	117	68.8
	≥70	53	31.20
Gender	Men	77	45.3
	Women	93	54.7
Education level	Illiterate	54	31.8
	Primary	50	29.4
	Intermediate	21	12.4
	High school	19	11.2
	University and higher	26	15.3
Marital status	Unmarried	49	28.8
	Married	121	71.2
Living status	Living with family	152	89.4
	Living Alone	18	10.6
Body mass index (kg/m^2^)		27.1 ± 2.9^˄^	
	Normal weight (18.5–24.9)	41	24.1
	Overweight (25.0–29.9)	129	75.9
Walking aid	Yes	33	19.4
	No	137	80.6
Falls in the past year	Yes	51	30
	No	119	70
Number of falling in the past year	Never	119	70
	Once	36	21.2
	More than one fall	15	8.8
FES-I scores (cut of point ≥ 30)		30.28 ± 13.2^˄^	
	Yes	79	46.5
	No	91	53.5
Injuries related to fall (n = 19)	Fractures	7	37
	Ankle swelling	1	5
	Bruises	11	58

All data are interpreted as frequency and % except data with ^˄^ are interpreted as mean ± SD.

% = percentage, N = number, FES-I = Falls Efficacy Scale-International.

Regarding health-related factors, 54.7% (n = 93) of the participants had vision problems, 54% (n = 92) had hypertension, 51% (n = 87) had diabetes mellitus, and 57.6% (n = 98) had 1 to 3 comorbidities. Symptoms of depression were reported by 32.4% (n = 55) of participants, and 19.4% (n = 33) reported their health as fair or poor. In addition, 104 participants (61%) reported joint pain, with 16.5% (n = 28) partially dependent on daily activities. Most of the participants (n = 135; 79.4%) took multiple medications. Regarding functional status, the mean grip strength for the dominant hand and TUG were 24.58 ± 7.99 kg, and 12.85 ± 2.84 seconds, respectively (Table 2).

**Table 2:** Health-related factors, medication use, depression, and physical function profile (n = 170).

Variables			Total (n = 170)	
			N	%
Health-related factors	Number of diseases	0	29	17.1
		1–3	98	57.6
		≥4	43	25.3
	Medications used	Yes	135	79.4
		No	35	20.6
	Medical conditions Reported as exclusion criteria	None	12	7.1
		Diabetes mellitus	87	51.2
		Hypertension	92	54.1
		Cardiovascular conditions	14	8.2
		Hyperlipidemia	70	41.2
		Osteoarthritis	49	28.8
		Osteoporosis	28	16.5
		Vision problems	93	54.7
		Kidney conditions	8	4.7
		Urinary incontinence	11	6.5
		COPD	9	5.3
	Depressive symptoms	Yes	55	32.4
		No	115	67.6
	Joint pain	UEP	13	7.6
		LEP	60	35.3
		LBP	31	18.2
	Health perception	Excellent	30	17.65
		Very good	46	27.05
		Good	61	35.88
		Fair	19	11.18
		Poor	14	8.24
Physical function	ADL	Partially dependent < 6	28	16.5
		Totally independent	142	83.5
	Grip strength (kg)	Total	24.58 ± 7.99	
		Men (n = 77)	30.8 ± 7.5	
		Women (n = 93)	19.6 ± 3.8	
	TUG (s)	Total	12.85 ± 2.84	
		Men	12.6 ± 2.7	
		Women	13.1 ± 2.9	

Data are presented as n (%), or mean ± SD.

% = percentage, ADL = activities of daily living, COPD = chronic obstruction pulmonary disease, LBP = low back pain, LEP = lower extremity pain, N = number, SD = standard deviation, TUG = times up and go test, UEP = upper extremity pain.

### 3.2. FES-I cutoff point

In the ROC analysis for FES-I to establish the cutoff point, it was found to be 30 (95% CI = 0.94–0.98, *P* < .05), yielding a sensitivity of 88.5% and specificity of 89.1% (Fig. S1, Supplemental Digital Content, https://links.lww.com/MD/P185). Participants with a score of <30 were categorized into the group without FOF, comprising 91 individuals. Meanwhile, those with a score of 30 or higher were placed in the FOF group, which consisted of 79 participants. The substantial agreement rate between the FES-I and a single FOF question was 88.8%. The percentage of FOF reported through the FES-I was 46.5%, whereas the single FOF question yielded a percentage of 45.9%. The kappa coefficient measuring the agreement between the single FOF question and FES-I was 0.78 (*P* < .001) (Table 3).

**Table 3:** Level of agreement between FOF and fall self-efficacy scale.

Fear of falling		Falls self-efficacy scale		Total
		No	Yes	
No fear	N (%)	82 (48.2)	10 (5.9)	92 (54.1)
Fear	N (%)	9 (5.3)	69 (40.6)	78 (45.9)
Total	N (%)	91 (53.5)	79 (46.5)	170 (100)

% = percentage, FOF = fear of falling, N = number.

### 3.3. Association between study variables with fear of falling using FES-I

Table 4 presents the results of the univariate analysis. Among individuals aged ≥ 70 years, the FOF was slightly higher than that in the 60 to 69 age group (49% vs 45.3%). However, this difference was not statistically significant (*P* = .65). Notably, women exhibited a significantly higher FOF rate than did men (54.8% vs 36.4%; *P* = .01). Moreover, participants who experienced falls showed a significantly higher FOF rate than non-fallers (68.6% vs 37%; *P* < .001). Among those using walking aids, a substantial majority expressed FOF (72.7%; *P* < .001).

**Table 4:** Sociodemographic characteristics, health-related factors, and physical function according to FOF using fall efficacy scale-international (n = 170).

Variables	N	Fear of falling				*P*-value
		No		Yes		
		N	%	N	%	
Age (yr)						
60–69	117	64	54.7	53	45.3	.65
≥70	53	27	50.9	26	49.1	
Gender						
Men	77	49	63.6	28	36.4	.01***
Women	93	42	45.2	51	54.8	
Education level						
Illiterate	54	25	46.3	29	53.7	.61
Primary	50	26	52.0	24	48.0	
Intermediate	21	12	57.1	9	42.9	
High school	19	12	63.2	7	36.8	
University and higher	26	16	61.5	10	38.5	
Marital status						
Unmarried	49	23	46.9	26	53.1	.22
Married	121	68	56.2	53	43.8	
Living status						
Alone	18	11	61.1	7	38.9	.49
With family or caregiver	152	80	52.6	72	47.4	
BMI						
Normal	41	23	56.1	18	43.9	.98
Overweight	129	73	56.6	56	43.4	
Falling history						
No	119	75	63.0	44	37.0	<.001*
Yes	51	16	31.4	35	68.6	
Walking aid						
No	137	82	59.9	55	40.1	<.001*
Cane	33	9	27.3	24	72.7	
Number of diseases						
0	29	20	69	9	31	<.001*
1–3	98	62	63.3	36	36.7	
≥4	43	9	20.9	34	79.1	
Number of medications						
0	35	26	74.3	9	25.7	<.001*
1–3	80	47	58.8	33	41.2	
≥4	55	18	32.7	37	67.3	
Depression						
No	115	75	65.2	40	34.8	<.001*
Yes	55	16	29.1	39	70.9	
UEP						
No	157	83	52.9	74	47.1	.54
Yes	13	8	61.5	5	38.5	
LEP						
No	110	69	62.7	41	37.3	.001*
Yes						
	60	22	36.7	38	63.3	
LBP						
No	139	78	56.1	61	43.9	.15
Yes	31	13	41.9	18	58.1	
Health perception						
Poor	14	3	21.4	11	78.6	.012**
Fair	19	7	36.8	12	63.2	
Good	61	33	54.1	28	45.9	
Very good	46	26	56.5	20	43.5	
Excellent	30	22	73.3	8	26.7	
Vison problems						
No	77	52	68	25	32	.001*
Yes	93	39	41.9	54	58.1	
ADL						
Partial dependence (<6)	28	5	17.9	23	82.1	<.001*
Independence (6)						
	142	86	60.6	56	39.4	
Grip strength	170	26.0 ± 7.9		23.2 ± 7.9		.02**
TUG	170	11.8 ± 1.9		14.1 ± 3.2		<.001*

Data are presented as n (%), or mean ± SD, chi-square test is employed for categorical variables.

%= percentage, ADL = activities of daily living, BMI = body mass index, FOF = Fear of falling, LBP = low back pain; LEP = lower extremity pain, N = number, SD = standard deviation, TUG = times up and go test, UEP = upper extremity pain.

* *P* < .001.

** *P* < .05.

*** *P* < .0.

Participants with a higher burden of comorbidities (≥4) and those with a higher rate of medication use (≥4) exhibited a significantly higher rate of FOF (79.1% and 67.3%, respectively; *P* < .001). Additionally, individuals experiencing depressive symptoms had a higher FOF (70.9%, *P* < .001).

Participants who perceived their health as excellent exhibited a significantly lower rate of FOF than those with poor health perception (26.7% vs 78.6%; *P* < .01). Moreover, most participants with vision problems reported a higher FOF rate (58.1%; *P* = .001). Additionally, participants experiencing LEP had a higher rate of FOF than those without pain (63.3% vs 37.3%, *P* = .001).

Considering daily activities, it was observed that a significant majority of partially dependent participants expressed FOF compared with their totally independent counterparts (82.1% vs 39.4%; *P* < .001). Additionally, the mean grip strength of participants with FOF was notably lower than that of those without FOF (23.2 ± 7.9 kg vs 26 ± 7.9 kg, *P* = .02). Furthermore, the mean TUG values for individuals with FOF and those without were 14.1 ± 3.2 seconds and 11.8 ± 1.9 seconds, respectively, revealing a significant difference (*P* < .001).

### 3.4. Factors predicting FOF

Based on the multivariate binary regression results presented in Table 5, the model revealed several significant associations. Participants with poor health perception were found to be 10 times more likely to experience FOF than those with excellent health perception (OR = 10.5, 95% CI = 1.26–87.73; *P* = .03). Women were 6 times more prone to FOF than were men (OR = 6.17, 95% CI = 1.57–24.14; *P* = .009). Participants with vision problems and a history of falling in the past year were 3 times more likely to have FOF than those without vision problems and non-fallers (OR = 3.81, 95% CI = 1.58–9.21; *P* = .003) and (OR = 3.29, 95% CI = 1.35–8.01; *P* = .009, respectively). Additionally, TUG value was identified as a predictor of FOF (OR = 1.38, 95% CI = 1.09–1.17; *P* = .007), while no medication use was more likely to have less FOF (OR = 0.03, 95% CI = 0–0.40, *P* = .007).

**Table 5:** Binary logistic regression model for risk factors of fear of falling.

	Categories	Odd ratio	95% CI for OR		*P*-value
			Lower	Upper	
Gender	Women	6.17	1.57	24.14	.009
	Men (ref)	1.00			
Falling history	Yes	3.29	1.35	8.01	.009
	No (ref)	1.00			
Number of medications	0	0.03	0.00	0.40	.007
	4+ (ref)	1.00			
Health perception	poor	10.51	1.26	87.73	.03
	Excellent (ref)	1.00			
Vision problems	Yes	3.81	1.58	9.21	.003
	No (ref)	1.00			
TUG		1.38	1.09	1.74	.007

CI = confidence interval, OR = odd ratio, (ref) = reference, TUG = times up and go test.

## 4. Discussion

This study aimed to identify the potential risk factors and predictors of FOF among older Saudi adults. This study found that FOF was a prevalent concern among older adults in Jeddah, affecting approximately 46.5% of the sample. However, our study’s FOF prevalence was lower than that in previous studies conducted in Egypt (64.4%)^[37]^ and Vietnam (64%),^[21]^ but it was more in line with the findings from studies in Thailand (50%)^[38]^ and Taiwan (53.4%).^[39]^ These variations in FOF prevalence across studies may be attributed to differences in population characteristics, such as history of falls, frailty, and environmental and cultural backgrounds.^[11]^

In this study, several factors were associated with FOF. These included sex, history of falls, health perception, vision problems, number of medications, and TUG test score. These variables are relatively straightforward to assess in a clinical setting and can effectively identify older individuals at an elevated risk of FOF.

The findings of our study indicate that a fall history is significantly associated with FOF. These results are in line with previous research^[12,40]^ and support a bidirectional relationship between falling and FOF,^[41]^ indicating that FOF can be considered a post-fall syndrome.^[42]^ However, Pohl et al^[43]^ did not support an association between FOF and future recurrent falls in community-dwelling people.

One of the key findings of this study was that women exhibited a higher susceptibility to FOF, which is in line with the findings of prior research.^[37,39,43]^ This could be attributed to factors such as the accelerated decline in bone mass among women, particularly following menopause, and the rapid loss of muscle mass due to reduced hormonal activity.^[44]^ Additionally, it is noteworthy that men may be more inclined to suppress their fear to avoid potential social stigma.^[20]^

This study failed to find an association between age and FOF, which is consistent with the results of previous studies.^[45,46]^ However, this result contradicts previous research by Saleh et al,^[34]^ Hoang et al,^[21]^ and Badrasawi et al.^[18]^ These discrepancies may be attributed to the fact that aging is not a precursor to FOF, and that various factors can result in FOF, such as psychological and physical characteristics.^[46]^

Our findings align with those of previous research that also did not confirm a significant association between education and FOF.^[47,48]^ However, Badrasawi et al^[18]^ and Saleh et al^[37]^ found a significant relationship between illiteracy and an increased FOF. Additionally, Bagley et al^[49]^ reported a significant relationship between years of education and the FOF. Education level alone may not fully explain FOF when other contributing factors are considered.

The findings of the present study affirm a negative association between FOF and declining functional ability, particularly ADL. Our results support the notion that older individuals who are more reliant on assistance for ADLs have diminished confidence in their physical abilities and tend to harbor more concerns about FOF. This finding is in agreement with previous research^[20,21]^ that identified a correlation between ADL scores and FOF.

In accordance with prior literature,^[18,21,27,50]^ our findings revealed that TUG test times were longer in older adults with FOF (14.1 ± 3.2 seconds) compared to their non-fearful counterparts (11.8 ± 1.9 seconds; *P* < .001). Furthermore, our study identified the TUG test as a predictor of FOF (*P* < .05) among older individuals. Prior research has established a TUG time ≥ 14 s as a risk factor for FOF.^[27]^ Other studies have suggested that a TUG time ≥ 12 s is indicative of a higher risk of falling.^[51]^ Consequently, it is reasonable to infer that older adults with FOF in our sample may face an increased risk of falls compared with their non-fearful counterparts.

In this study, there was a significant difference in grip strength between older individuals with and without a FOF (*P* < .02). However, grip strength was not a predictor of FOF. This finding is consistent with the results of previous studies.^[6,50]^ In the elderly, FOF can initiate a detrimental cycle of reduced physical activity, which subsequently leads to a decline in muscle strength.^[52]^

Our findings align with previous research that identified health perception as a potent indicator of health status and a risk factor for FOF among older adults.^[21,38]^ The perception of poor health may result in fatigue and weakness, affecting the confidence of older adults in carrying out their daily activities. This, in turn, may increase the risk of activity limitations attributed to FOF.^[21,29]^

Our study revealed a significant association between visual impairment and FOF, which is consistent with previous research.^[47]^ In a systematic review, visual problems were identified as a relatively weak yet notable factor associated with FOF within the context of previously defined risk factors.^[17]^ This may be due to cultural and environmental differences or differences in FOF measurements.

Consistent with prior research, our results confirmed that the number of diseases and medications were significantly associated with FOF.^[37,38]^ This might be attributed to the potential side effects of medications, such as drowsiness and elevated risk of inappropriate drug interactions, resulting in balance and orientation problems.^[53]^ Furthermore, present study revealed that older adults with depression were more likely to experience FOF which aligns with prior research.^[12,17,21,54]^ Additionally, depression typically accompanies reduced energy levels and increased fatigue, leading individuals to have less faith in their physical abilities, diminished self-confidence and heightened concerns about falling.^[12,55]^

In this study, UEP and low back pain did not exhibit a significant association with FOF, in contrast to LEP, which showed a significant correlation with FOF. Interestingly, Tomita et al^[6]^ reported findings that differed from ours, as they observed associations between all 3 pain variables and FOF. This disparity could potentially be attributed to differences in study participants, given that their cohort consisted exclusively of women, among whom joint pain tended to be more prevalent. Women face a higher risk of numerous common pain conditions, particularly joint pain resulting from factors such as osteoporosis and joint inflammation.^[56]^ Furthermore, variations in the methodologies employed in the 2 studies may have contributed to discordant results. LEP is theorized to be linked to FOF because it is hypothesized that pain and musculoskeletal symptoms associated with conditions such as osteoarthritis (OA) lead to reduced usage of the affected extremity, muscle weakness, impaired balance, and diminished functional performance, consequently increasing the risk of falls.^[57]^ This sense of insecurity stemming from pain-induced limitations may contribute to the development of FOF.

This study has several strengths. First, this is one of the few studies conducted to explore FOF and its associated risk factors among older Saudi adults. Second, it established a specific cutoff point for the Arabic version of the FES-I (Falls Efficacy Scale-International), enhancing the utility of this assessment tool. Finally, 16 risk factors were assessed to cover the most reported risk factors, as reported in the literature review. However, this study had several limitations that should be considered when interpreting the results. First, the cross-sectional design employed in this study did not provide a basis for establishing causal relationships between variables. Therefore, further research utilizing different study designs is required to confirm the relationship between the reported variables and FOF. Second, the generalizability of the study’s findings may be limited because of the recruitment of participants through convenience sampling from Jeddah. It is advisable to replicate this study with larger and more diverse sample sizes, possibly incorporating longitudinal research methods, and following up the information for a more comprehensive understanding of FOF dynamics over time. Furthermore, investigation of the association between other factors, such as frailty, anxiety, and FOF, in Saudi community-dwelling older adults is recommended. Additionally, although the current study used hand grip strength and the Timed Up and Go (TUG) test solely to assess physical function in relation to FOF, these measures are also indicative of sarcopenia. Therefore, further research is required to investigate their association with FOF in the context of sarcopenia.

Implications for health practice and policies: this study identified key risk factors for FOF in community-dwelling older adults. Therefore. incorporating FOF management in standard geriatric care including comprehensive assessments, fall prevention education, and community-based social support, to improve health and reduce fall-related injuries.

## 5. Conclusion

FOF is a prevalent issue among older adults in Saudi Arabia. Several factors were identified as predictors of FOF, including sex (being a woman), a history of falls, performance on the TUG test, poor health perception, and vision problems. No medication use was more likely to result in a lower FOF. These findings highlight the importance of FOF assessment and the development of appropriate rehabilitation interventions. Encouraging older adults to engage in regular physical activity and incorporating sensory impairment screening into routine care can be instrumental in mitigating the FOF.

## Author contributions

**Conceptualization:** Rehab Abdulrhman Al Harbi, Rehab F.M. Gwada.

**Data curation:** Rehab Abdulrhman Al Harbi.

**Formal analysis:** Rehab Abdulrhman Al Harbi, Mohammed T.A. Omar, Rehab F.M. Gwada.

**Investigation:** Rehab Abdulrhman Al Harbi.

**Methodology:** Rehab Abdulrhman Al Harbi, Mohammed T.A. Omar, Samiha Mohamed Ibrahim Abd Elkader, Saad A. Alhammad, Rehab F.M. Gwada.

**Supervision:** Rehab F.M. Gwada.

**Validation:** Rehab F.M. Gwada.

**Visualization:** Rehab F.M. Gwada.

**Writing – original draft:** Rehab Abdulrhman Al Harbi, Mohammed T.A. Omar, Samiha Mohamed Ibrahim Abd Elkader, Saad A. Alhammad, Rehab F.M. Gwada.

**Writing – review & editing:** Mohammed T.A. Omar, Samiha Mohamed Ibrahim Abd Elkader, Saad A. Alhammad, Rehab F.M. Gwada.

## Supplementary Material


