# Supplementary material for: Factors associated with fear of falling among Saudi community-dwelling older adults: A cross-sectional study
Source: Medicine (Baltimore). 2025 Jun 13;104(24):e42864. doi: 10.1097/MD.0000000000042864 (PMC12173336; doi:10.1097/MD.0000000000042864)
Supplement: Supplementary file 1 [file medi-104-e42864-s001.docx]

**Figure S1:** ROC curve for identifying cut-off point of falls self-efficacy
